# Betahistine’s Neuroprotective Actions against Lipopolysaccharide-Induced Neurotoxicity: Insights from Experimental and Computational Studies

**DOI:** 10.3390/brainsci14090876

**Published:** 2024-08-29

**Authors:** Vasudevan Mani, Minhajul Arfeen

**Affiliations:** 1 Department of Pharmacology and Toxicology, College of Pharmacy, Qassim University, Buraydah 51452, Saudi Arabia; 2 Department of Medicinal Chemistry and Pharmacognosy, College of Pharmacy, Qassim University, Buraydah 51452, Saudi Arabia; m.arfeen@qu.edu.sa

**Keywords:** betahistine, lipopolysaccharide, cognitive functions, neuroinflammation, mitochondria, apoptosis, antioxidants, molecular docking simulation

## Abstract

Histamine H_3_ receptor (H_3_R) antagonists, such as betahistine (BHTE), have shown significant potential in treating central nervous system (CNS) disorders due to their neuroprotective properties. This study investigated BHTE’s effects on lipopolysaccharide (LPS)-induced neurotoxicity, which is associated with neuroinflammation and neurodegeneration. Rats were divided into groups and pre-treated with BHTE (5 or 10 mg/kg, p.o.) for 30 days, followed by LPS administration (1 mg/kg, i.p.) for 4 consecutive days to induce neurotoxicity. LPS exposure resulted in cognitive impairment, as evidenced by performance deficits in maze tests, and a significant reduction in brain acetylcholine (ACh) levels. Additionally, LPS led to increased neuroinflammation, oxidative stress, mitochondrial dysfunction, and apoptosis. Pre-treatment with BHTE effectively counteracted these effects, improving cognitive performance and restoring ACh levels. BHTE significantly reduced LPS-induced increases in pro-inflammatory markers (COX-2, TNF-α, and IL-6) while enhancing anti-inflammatory cytokines (IL-10 and TGF-β1). Furthermore, BHTE improved mitochondrial function by increasing enzyme levels (MRCC-I, II, and IV) and boosted anti-apoptotic (Bcl-2) and antioxidant defenses (GSH and catalase). BHTE also reduced apoptosis markers, including pro-apoptotic protein caspase-3, and oxidative stress marker malondialdehyde (MDA). Molecular modeling studies revealed that BHTE effectively binds to key enzymes involved in neuroinflammation and apoptosis (AChE, COX-2, and caspase-3), with binding free energies between 4 and 5 kcal/mol, interacting with critical residues. These findings underscore BHTE’s multifaceted neuroprotective effects against LPS-induced neurotoxicity, offering potential therapeutic avenues for managing neuroinflammation and related neurodegenerative disorders.

## 1. Introduction

One or more disease-specific mechanisms that trigger inflammatory responses contribute to neurodegenerative disorders. Understanding the underlying molecular causes of these disorders and finding neuroprotective treatments are crucial needs because they represent a significant public health concern. Neuroinflammation is considered a natural immunological response of the brain that speeds up the onset of the neuronal degenerative process in most neurodegenerative disorders, including Alzheimer’s disease (AD) [1]. Moreover, the majority of brain cells, including neurons, microglia, and macroglia, are also affected by inflammatory insults, and they are also linked to some degree of tissue damage, such as the loss of myelin sheaths or axons [2]. Particularly, microglia play a crucial role in the neuronal inflammation process; these are activated through many factors like pathogenic infections, tissue damage, oxidative stress, a variety of neurotoxins, and any injuries. Numerous mediators, including chemokines, pro-inflammatory cytokines, prostanoids, cyclooxygenase (COX) enzymes, nitric oxide, apoptosis factors, and ROS generated by microglia, are crucial for maintaining the inflammatory response in neurons [1,3].

Since COX enzymes are considered rate-limiting enzymes to generate prostaglandins (PGs) from arachidonic acid. The isomers of both COX enzymes (COX-1 and COX-2) are reported in neurodegeneration through the neuroinflammatory process. Specifically, neurons predominantly exhibit the expression of COX-2, and this expression disrupts both synapse functioning and the memory process [3]. Moreover, the expression of pro-inflammatory cytokines such as interleukin (IL)-1β and tumor necrosis factor (TNF)-α can induce the biosynthesis of PGs via the activation of COX enzymes. Furthermore, inhibition of the IL-6 receptor decreased the cell proliferation produced by IL-1β, TNF-α, and COX-2-derived PGs [4]. Conversely, the immune response’s pro-inflammatory cytokines are regulated by a group of immunoregulatory molecules referred to as anti-inflammatory cytokines. Among these, IL-10 serves as a robust anti-inflammatory cytokine, effectively suppressing the synthesis of pro-inflammatory cytokines, such as TNF-α, IL-6, and IL-1, by activated macrophages [5]. Furthermore, isomers of transforming growth factor (TGF)-β cytokines prevent the synthesis of TNF-α, IL-1, IL-2, and IL-6 by inhibiting macrophage and T helper 1 cell activities [6].

Neuroinflammation may directly influence neuronal death by mediating numerous pro-apoptotic pathways through excessive activation of signaling molecules during inflammatory process [7]. In focus, the binding of TNF-α to its specific receptor tumor necrosis factor receptor 1 (TNFR1) causes a rapid apoptosis process in neurons, which is mediated through the caspase-3 and caspase-8 pathways [8,9]. In extension, inflammation-related DNA damage and oxidative stress result in increasing macrophage and microglial production of ROS in the brain. It is widely known that ROS have a high degree of reactivity and are directly harmful to biological macromolecules. They can also cause inflammation by activating a variety of genes that control the signaling cascades of inflammation [3]. Additionally, mitochondria are essential as cellular sources of redox signaling molecules, including superoxide anion radicals (O_2_^•−^) and hydrogen peroxide (H_2_O_2_), produced through oxidative metabolism. Complexes I and II of the mitochondrial electron transport chain (mETC) generate O_2_^•−^/H_2_O_2_ in the mitochondrial matrix, while complex III releases these reactive species into the cristae lumen and intermembrane space. [10]. Furthermore, complex IV plays a role in converting O_2_ to H_2_O, overseeing the final step of the mECT [11].

An endotoxin, LPS derived from Gram-negative bacteria produces systemic inflammation by activating Toll-like receptor (TLR)-4 signaling pathways. TLRs are a subclass of pattern recognition receptors (PRRs) that are largely present in astrocytes and microglia of the CNS. They can recognize various hostile signals and promptly respond by inducing inflammation [12]. In rodents, LPS by systemic injection leads to inducing upregulations in pro-inflammatory cytokines like TNF-α, IL-6, and IL-1β; additionally, elevation on nuclear factor kappa B (NF-κB) involves further stimulation of other inducible enzymes, including inducible nitric oxide synthase (iNOS) and COX-2. The production of all the above inflammatory mediators induces neuroinflammation, particularly in the cortex and hippocampal areas affecting cognitive functioning [3,13,14,15]. Furthermore, both acute and chronic exposure to LPS resulted in apoptotic neuronal death in NE-4C mouse neural stem cells [16]. Under continuous administration, LPS induced both TNF-α- and nitric oxide-mediated apoptosis in bone marrow-derived macrophages [17].

Betahistine (BHTE) is a structural analog of histamine neurotransmitter and is frequently prescribed for vestibular vertigo; additionally, it is also particularly effective in treating the symptoms of Ménière’s disease [18]. It acts as a potent antagonist of H_3_R and a weak agonist of H_1_R, and both receptors activate G-proteins on the neuronal cell membrane. In focus, H_3_R originates in neuronal pre-synaptic and acts as auto- and hetero-receptors, they alter the releases of neurotransmitters, including histamine, acetylcholine, and serotonin. Most of these neurotransmitters have a major role in various cognitive functions [19]. Since histamine has been related to a variety of brain illnesses, including AD, schizophrenia, and epilepsy, the histaminergic system is an appropriate therapeutic target for treating these conditions [20,21]. Several studies have supported that inverse agonists of H_3_R resulted in the enhancement of learning and memory performance [14,19,22,23].

Recently, the administration of BHTE alleviated the stimulation of a generalized class of tonic clonic seizures and reduced the intensity of forelimb clonic seizures in pentylenetetrazole (PTZ)-induced kindling mice. Also, the administration improved memory diminishing and depression behavior by protecting the neuronal injury in the hippocampal and cerebral cortex from PTZ toxicity [24]. The BHTE’s protective effect against PTZ-challenged neuronal degeneration in the cortex and hippocampus was also evidenced by a decline in caspase-3, a microglia marker protein [ionized calcium-binding adaptor molecule 1 (IBA1)], and glial fibrillary acidic protein expression while improving synaptophysin levels [25]. Early on, due to improved blood flow and the vasodilatory effects of BHTE, the patient with vertebrobasilar insufficiency and dementia showed significant neurologic and neuropsychological improvement [26]. Moreover, it activated the neurons in the perirhinal cortex area by enhancing the release of histamine and improved long-term memory in mice [27]. Our results demonstrated that continuous 28-day administration of BHTE mitigated chemotherapy-induced cognitive impairment in a doxorubicin-induced chemobrain model by enhancing cholinergic function and reducing neuronal inflammation [28]. Based on the above literature, our study aimed at exploring the protective mechanisms of BHTE on LPS-challenged memory dysfunctions and neurotoxicity in rat models.

## 2. Materials and Methods

### 2.1. Animals

For our experimental procedures, a total of thirty adult male albino rats were acquired from the animal facilities located at the College of Pharmacy, Qassim University, KSA. These rats, aged between eleven to twelve weeks and weighing 150–170 g, were divided into five groups, with each group comprising six rats. They were housed in cages with three animals per cage, maintained under a 12 h light and 12 h dark cycle, and provided with free access to food and water. Prior to initiating drug treatment, the rats underwent a one-week acclimatization period to adjust to the laboratory environment. Ethical approval for the use of animals in this study was obtained from the Committee of Health Research Ethics, Deanship of Scientific Research, Qassim University, under research number 21-16-22 and grant number 2023-SDG-1-HMSRC-35650.

### 2.2. Treatment Groups and Experiment Schedule

**Figure 1:**
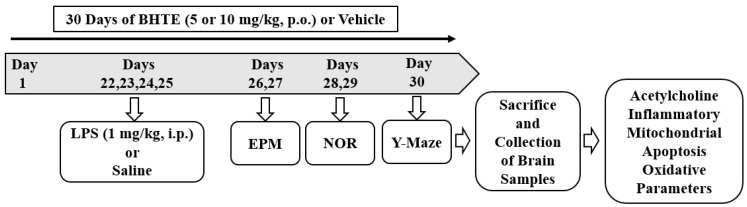
Timeline of the in vivo experiments: Rats were divided into groups and given either a vehicle or betahistine (BHTE) orally for a duration of 30 days. Four doses of vehicle or lipopolysaccharide (LPS; 1 mg/kg, i.p.; days 22, 23, 24, and 25) for inducing neurotoxicity. Elevated plus-maze (EPM; days 26 and 27), novel object recognition (NOR; days 28 and 29), and Y-Maze (day 30) were conducted for cognitive assessments. On the 30th day, following the completion of memory tests, all animals were euthanized, and brain tissues were collected for ELISA analysis.

BHTE hydrochloride and LPS from Escherichia coli (O111:B4) were secured from Sigma-Aldrich Co (St. Louis, MO, USA) and dissolved in normal saline (NS; 0.09% *w*/*v*). This study involved dividing rats into five groups to investigate the effects of BHTE on LPS-induced neurotoxicity. In the control group (Group 1), animals received oral administration of NS (0.1 mL/100 mg) from day 1 to day 30 along with intraperitoneal (i.p.) injections of NS (1 mg/kg; 4 doses) from day 22 to 25 to ensure standardized treatment (Figure 1). Group 2, named BHTE10, received oral BHTE at a dosage of 10 mg/kg from day 1 to day 30 along with i.p. injections of NS (1 mg/kg; 4 doses) from day 22 to 25. Group 3, identified as LPS, received oral NS from day 1 to day 30 and i.p. injections of LPS (1 mg/kg; 4 doses) were administered from day 22 to 25. Groups 4 and 5, denoted as BHTE5 + LPS and BHTE10 + LPS, respectively, were given oral betahistine at doses of 5 and 10 mg/kg, respectively, from day 1 to day 30, alongside i.p. injections of LPS (1 mg/kg; 4 doses) from day 22 to 25. The selection of BHTE and LPS doses is based on prior research [25,28,29]. The evaluation of cognitive performance involved a series of tests. On days 26 and 27, animals underwent elevated plus-maze (EPM) procedures for training and retention, respectively. Days 28 and 29 included novel object recognition (NOR) tests for training and a subsequent test session. Both sessions (training and test) of the Y-maze test were conducted on day 30. Following the behavioral assessments, brain tissues were utilized exclusively for ELISA tests.

### 2.3. Elevated Plus-Maze (EPM) Test

The EPM is a commonly employed behavioral model applied to evaluate memory in rodents. This maze configuration consists of two enclosed and open arms each, with the entire apparatus elevated 50 cm above ground level. Rodents, displaying a natural aversion to exposed and elevated environments, tend to favor the enclosed arms for exploration and prolonged stay. In the experimental setup, memory performance in rats was assessed using transfer latency (TL), which represents “the time taken by rats to transition from any open arm to an enclosed arm”. TL assessments were carried out on both the training day (26th day) and the experimental day (27th day). During the training phase, rats were allotted a two-minute exploration period within the maze. Following this exploration, memory retention is evaluated after a 24 h delay. These procedural details have been expounded upon by early reports [29,30].

### 2.4. Novel Object Recognition (NOR) Test

The NOR test is a widely utilized method for assessing rodents’ cognitive abilities, particularly their recognition memory, across diverse experimental CNS models. As previously mentioned, this task was conducted within a wooden box measuring 80 × 60 × 40 cm, housing two dissimilar objects of similar size and height [29,30]. Among these objects, two cylindrical boxes, labeled FO1 and FO2, were identified as familiar objects, while a rectangular box served as the novel object (NO). The experimental procedure was structured into three distinct sessions: habituation (28th day), training, and testing phases (29th day). During the habituation phase, each rat was allotted five minutes to freely explore the empty box, allowing for them to familiarize themselves with the environment. Following a 24 h interval, during the training session, rats were introduced into the box containing two identical objects (FO1 and FO2, positioned symmetrically on both the right and left sides) and permitted five minutes to explore both objects. Subsequently, three hours after the training session, the test phase commenced. This phase replicated the training session’s procedure, with the exception of substituting one FO with the NO. Exploration times for each object (ETFO and ETNO) were recorded using a webcam, defining exploration as “directing the nose toward the object at a distance of equal to or less than 2 cm”. Furthermore, the percentage discrimination index (DI%) was calculated utilizing the formula ((ETNO − ETFO)/Total ET) × 100), aimed at elucidating the animals’ capacity for exploring novelty versus familiarity.

### 2.5. Y-Maze Test

The evaluation of rats’ spatial memory and their tendency to explore new environments was conducted using the Y-maze test. This test utilized a wooden Y-maze comprising three arms (50 × 10 × 30 m^3^; L × W × H) positioned at 120-degree angles to each other. At the end of each arm, there was an image with various patterns, and the maze was placed on the floor. During the training session, which took place on the 30th day, one arm, designated as the novel arm, was kept closed while the rats were allowed to freely explore the other two arms for 5 min. After a three-hour interval, the novel arm was then opened, and the rats were given an additional 5 min session to explore, in a test session. The number of entries into both the known arm (NEKA) and novel arm (NENA) was noted, and subsequently, the percentage of total time spent in the novel arm (TSNA%) was computed for each individual rat [29,30].

### 2.6. Preparation of Brain Homogenate

On the 30th day of drug treatment, after completing the maze experiments, all the rats were euthanized by cervical decapitation under anesthesia (using ketamine at 100 mg/kg and xylazine at 10 mg/kg; i.p.). Following extraction from the skull, all brain tissues were carefully minced into small pieces and thoroughly rinsed in ice-cold phosphate-buffered saline (PBS, pH 7.4) to remove any excess blood. The tissue fragments were then weighed and homogenized in PBS at a ratio of 1 g of tissue to 9 mL of PBS using a glass homogenizer on ice. The resulting homogenates were subsequently used for ELISA analysis to target specific markers. The quantification of total protein in each homogenized brain sample was carried out using the biuret colorimetric method, as per the protocol provided by Crescent Diagnostics, located in Jeddah, Saudi Arabia.

### 2.7. Estimation of Acetylcholine (ACh)

ACh levels in brain homogenate were estimated using an ELISA kit obtained from MyBioSource (MBS728879; MyBioSources Inc., San Diego, CA, USA). The assay employed a competitive enzyme immunoassay method, utilizing a polyclonal anti-ACh antibody and an ACh-HRP conjugate. Initially, 100 µL of either the standard or homogenate was added to each designated well. Finally, the intensity of color changes corresponding to ACh quantification was measured spectrophotometrically at 450 nm using a microplate reader (BioTek Instruments, Santa Clara, CA, USA).

### 2.8. Neuroinflammatory Markers

Inflammatory biomarkers such as COX-2 (MBS266603), TNF-α (MBS162068), IL-6 (MBS2701082), IL-10 (MBS702776), and TGFβ-1 (MBS824788) were selected for analysis. The assays were conducted using specific rat ELISA kits obtained from MyBioSources (MyBioSources Inc., San Diego, CA, USA). The estimation principle was based on the interaction between the specific antigen and a biotinylated detection antibody, along with horseradish peroxidase–streptavidin (SABC), which caused a color change in the solution. The concentration was determined by comparing the spectrophotometric measurements at 450 nm with a standard curve.

### 2.9. Mitochondrial Respiratory Chain Complexes (MRCCs) Enzymes

To comprehend the pivotal role of mitochondrial function, the levels of selected MRCCs were assessed in brain homogenate. Specifically, rat MRCC-I (MBS7255374), MRCC-III (MBS2606461), and MRCC-IV (MBS3808665) ELISA kits from MyBioSources (MyBioSources Inc., San Diego, CA, USA) were employed to quantify the relevant enzymes.

### 2.10. Apoptotic Proteins

The levels of apoptotic proteins were evaluated in this study. An anti-apoptotic protein, B-cell lymphoma-2 (Bcl-2; MBS2515143), was assessed alongside two pro-apoptotic proteins: Bcl2 associated X protein (Bax; MBS165136) and caspase-3 (MBS763727). Rat-specific ELISA kits obtained from MyBioSources (MyBioSources Inc., San Diego, CA, USA) were employed for these measurements. The assays were conducted according to the manufacturer’s instructions, and the absorbance readings were recorded at 450 nm using a Microplate Reader.

### 2.11. Oxidative-Related Markers

Malondialdehyde (MDA; MBS268427), an oxidative marker, as well as antioxidant markers including catalase (CAT; MBS2704433) and glutathione (GSH; MBS265966), were measured using ELISA assay kits specific to rats, sourced from MyBioSources (MyBioSources Inc., San Diego, CA, USA). The color development was analyzed at 450 nm with a Microplate Reader (BioTek Instruments, Santa Clara, CA, USA).

### 2.12. Statistical Analysis

The results were expressed as mean values with the standard error of the mean (SEM). Statistical analysis included performing one-way ANOVA on the data, followed by the Tukey–Kramer post-hoc test to identify significant differences between groups. In the NOR test, an unpaired *t*-test was used to compare the ETFO and ETNO groups. All statistical analyses were conducted using GraphPad version 9.0 (GraphPad Software Inc., San Diego, CA, USA), with significance set at *p* < 0.05.

### 2.13. Molecular Docking

Molecular docking was conducted by utilizing AutoDock Vina (AutoDock Vina, La Jolla, CA, USA). The protein structures employed were 1DX6, 5IKR, and 1GFW for AChE, COX-2, and caspase-3, respectively. AutoDock tools bundled with MGL tools (version 1.5.6, La Jolla, CA, USA) was used to prepare input files. The crystal structures for molecular docking were obtained from the Protein Data Bank (PDB). The three-dimensional structure of betahistine in SDF format was obtained from the PubChem database. Heteroatoms, water molecules, and any extra chains if present were removed; polar hydrogens, missing atoms, and Kollman charges were added, followed by the conversion of protein molecules to pdbqt format. The downloaded molecule of BHTE was converted to pdbqt format after minimization using the universal force field, defining torsions and adding Gasteiger charges. The grids were specified by setting the co-complexed small molecule at the center. Configuration files containing information about the receptor, ligands, box size, and the coordinates for the center of the box were created. The docking results were generated in terms of kcal/mol. Binding mode analysis was performed by considering top three low-energy conformations. The conformation displaying interactions with key amino acids was included for discussion.

## 3. Results

### 3.1. BHTE Reversed LPS-Induced Spatial Memory Impairment in EPM Test

The current study utilized the EPM task on both day 26 and day 27 to assess learning capability and memory retention capability of animals during BHTE treatment. Here, the transfer latency (TL) values were measured as a memory parameter and shorter TL values were reflected in the higher learning ability and retention capability.

**Figure 2:**
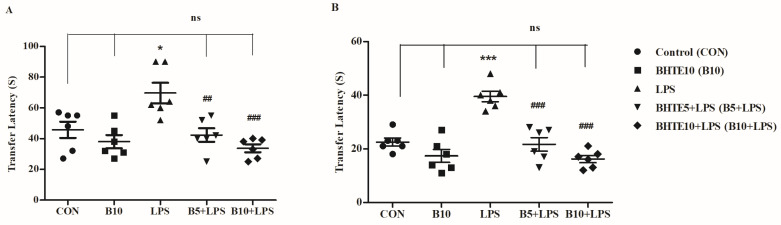
Impact of BHTE on the retention of TL (s) on (**A**) day 1 and (**B**) day 2 in rats induced with LPS in EPM test. The results are reported as the mean ± SEM, with a sample size of n = 6. One-way ANOVA [F(4,25) = 8.501, *p* < 0.001 for day 1; and F(4,25) = 21.63, *p* < 0.001 for day 2], followed by Tukey–Kramer multiple comparisons test. * *p* < 0.05 and *** *p* < 0.001 vs. control; ns—not significant vs. control; ## *p* < 0.01 and ### *p* < 0.001 vs. LPS.

Initially, a one-way ANOVA was conducted to analyze the overall changes in TL among all treatment groups, revealing significant alterations [F(4,25) = 8.501, *p* < 0.001 for day 1, and F(4,25) = 21.63, *p* < 0.001 for day 2] in TL values (Figure 2). Focused on day 1, peripheral injections of LPS at four different doses over four consecutive days (days 22–25 of the treatment schedule) prolonged TL by 34%, with a value of 69.67 ± 6.642 S (*p* < 0.05), compared to the control value of 45.67 ± 5.302 S (Figure 2A). Treatment with BHTE improved the animals’ learning ability by reducing TL to 42.33 ± 4.341 S (*p* < 0.01; 39% reduction) and 33.67 ± 2.629 S (*p* < 0.001; 51% reduction) at doses of 5 mg/kg and 10 mg/kg, respectively. Additionally, BHTE alone at a dose of 10 mg/kg (p.o.) resulted in a TL value of 38.00 ± 4.258 S.

On day 2, LPS treatment significantly prolonged TL by 43% (39.50 ± 1.990 S) compared to control rats (22.50 ± 1.500 S, *p* < 0.001) (Figure 2B). Subsequent treatment with BHTE effectively reversed the LPS-induced increase in TL. Specifically, doses of 5 mg/kg (21.67 ± 2.525 S; 45% reduction) and 10 mg/kg (16.17 ± 1.352 S; 59% reduction) significantly reduced TL compared to LPS treatment (*p* < 0.001). Moreover, the higher dose of BHTE (10 mg/kg, p.o.) alone did not produce significant changes in TL values (17.33 ± 2.431 S) compared to the control group.

### 3.2. BHTE Reversed LPS-Induced Recognition Memory Deficits in NOR Test

**Figure 3:**
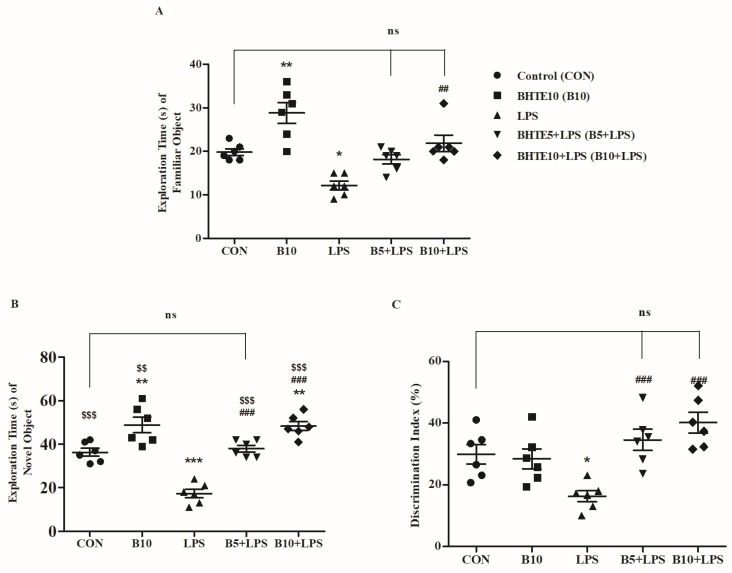
The effects of BHTE on (**A**) ETFO, (**B**) ETNO, and (**C**) DI% in LPS-induced rats during the test session in NOR test. The results are reported as the mean ± SEM, with a sample size of n = 6. One-way ANOVA [F(4,25) = 14.95, *p* < 0.001 for ETFO; F(4,25) = 30.02, *p* < 0.001 for ETNO; and F(4,25) = 10.77, *p* < 0.001 for DI%], followed by Tukey–Kramer multiple comparisons test for within-group comparisons. * *p* < 0.05, ** *p* < 0.01, and *** *p* < 0.001 vs. control; ns—not significant vs. control; ## *p* < 0.01 and ### *p* < 0.001 vs. LPS. The student’s unpaired *t*-test computed to compare ETFO vs. ETNO, $$ *p* < 0.01 and $$$ *p* < 0.001 vs. corresponding ETFO group.

The NOR test is commonly used to evaluate the recognition memory of rodents with respect to specific treatments. In this study, two different objects were used to assess the recognition memory of the rats. Three parameters were analyzed such as exploration time of familiar object (ETFO) as well as novel object (ETNO) and percentage discrimination index (DI%) during the maze experiment (Figure 3).

Figure 3A illustrates the effect of BHTE and LPS on the ETFO in rats. A significant variation was observed among all treatments [F(4,25) = 14.95, *p* < 0.001]. When performing individual comparisons between treatments, LPS resulted in a 38% reduction (*p* < 0.05) in ETFO to 12.17 ± 1.014 S, compared to control rats with an ETFO of 19.83 ± 0.7923 S. In contrast, BHTE10 treatment significantly increased ETFO by 44% (*p* < 0.01) to 21.83 ± 1.887 S compared to LPS treatment. BHTE5 treatment did not show a considerable alteration in ETFO (18.17 ± 1.078 S) compared to both the control and LPS challenge. Additionally, the treatment of BHTE10 in normal rats resulted in an ETFO of 28.83 ± 2.414 S.

Additionally, the effect of BHTE and LPS on the ETNO is displayed in Figure 3B. An initial one-way ANOVA analysis revealed a notable variation [F(4,25) = 30.02, *p* < 0.001] between all treatments. LPS-treated rats exhibited a marked decline of 52% (*p* < 0.001) in ETNO to 17.33 ± 1.978 S, compared to control rats with an ETNO of 36.33 ± 1.856 S. Co-administration with BHTE5 significantly increased ETNO to 38.00 ± 1.549 S (54%; *p* < 0.001) and BHTE10 further increased ETNO to 48.33 ± 2.108 S (64%; *p* < 0.001) in the LPS-treated rats. Moreover, BHTE10 alone improved ETNO to 48.83 ± 2.108 S. Furthermore, a comparison analysis between ETFO and ETNO showed that, except for the LPS-treated rats, all other groups exhibited significant improvements in ETNO (*p* < 0.01 for BHTE10 and *p* < 0.001 for the remaining treatments).

The DI% was calculated to understand the influence of BHTE and LPS on the rats’ ability to discriminate between the FO and the NO (Figure 3C). The analysis of the results among the treatments revealed a significant change [F(4,25) = 10.77, *p* < 0.001]. Consistent with the previous results, LPS-treated rats showed a lower DI% of 16.34 ± 1.827% compared to the control group’s performance of 29.86 ± 3.158% (*p* < 0.05). Conversely, treatments with LPS + BHTE5 (34.55 ± 3.436%) and LPS + BHTE10 (40.13 ± 3.353%) exhibited significant improvements (*p* < 0.001) in the rats’ discrimination ability. Additionally, rats treated with BHTE10 alone recorded a DI% of 28.42 ± 3.283%.

### 3.3. BHTE Attenuated LPS-Induced Spatial Recognition Memory Deficits in the Y-Maze Test

**Figure 4:**
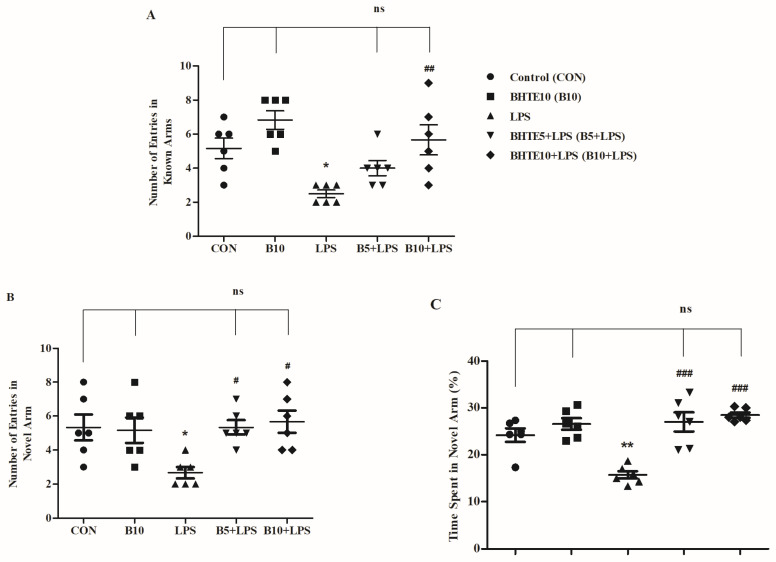
The impact of BHTE on (**A**) NEKA, (**B**) NENA, and (**C**) TSNA% during the test session of LPS-induced rats in Y-maze. The results are reported as the mean ± SEM, with a sample size of n = 6. One-way ANOVA [F(4,25) = 8.127, *p* < 0.001 for NEKA; F(4,25) = 4.006, *p* < 0.05 for NENA; F(4,25) = 14.65, *p* < 0.001 for TSNA%], followed by Tukey–Kramer multiple comparisons test. * *p* < 0.05, and ** *p* < 0.01 vs. control; ns—not significant vs. control; # *p* < 0.05, ## *p* < 0.01 and ### *p* < 0.001 vs. LPS.

The Y-maze task was employed to explain the influence of BHTE and LPS treatment on the rats’ innate tendency to explore a novel environment, reflecting their spatial recognition memory. In the test session, conducted three hours after the training session, the rats were allowed access to all three arms of the maze: two familiar arms and one novel arm. Three parameters were targeted: number of entries into known arm (NEKA) as well as novel arm (NENA), and percentage of total time spent in the novel arm (TSNA%) (Figure 4).

Considering the results for NEKA with BHTE and LPS treatments, a significant variation was observed [F(4,25) = 8.127, *p* < 0.001] in rats (Figure 4A). Compared to the control NEKA value of 5.167 ± 0.6009, four consecutive injections of LPS reduced NEKA by 51% (*p* < 0.05) to 2.500 ± 0.2239. However, continuous administration of BHTE10 for thirty days along with LPS injections increased NEKA by 55% (*p* < 0.05), with the values reaching 5.667 ± 0.8819. Additionally, the BHTE5 + LPS treatment improved NEKA to 4.000 ± 0.4472, although this increase was not statistically significant. Rats treated with BHTE10 alone attained NEKA levels of 6.833 ± 0.5426.

A similar pattern of results was found in the NENA performance. Comparison among all the treatments showed the impact of BHTE and LPS on rats [F(4,25) = 4.006, *p* < 0.05] (Figure 4B). Similar to NEKA, NENA performance declined by 49% (*p* < 0.05) following LPS treatments, with values dropping to 2.667 ± 0.3333 compared to the control treatment at 5.333 ± 0.7601. Co-administration of both dose levels, BHTE5 + LPS (5.333 ± 0.4216; 49%) and BHTE10 + LPS (5.667 ± 0.6667; 52%), significantly reversed (*p* < 0.05) the decline in NENA seen with LPS treatment alone. The BHTE10 treatment alone showed a similar level of performance (5.167 ± 0.7491) as the control.

Regarding the results of TSNA% among the treatments, there was a considerable alteration [F(4,25) = 14.65, *p* < 0.001] after treatment with BHTE and LPS (Figure 4C). LPS treatment significantly reduced TSNA% (*p* < 0.01) to 15.72 ± 0.7870% from the control value of 24.15 ± 1.458%. However, the administration of BHTE5 + LPS stabilized TSNA% by rats to 27.00 ± 2.049% (*p* < 0.001). Additionally, the higher dose of BHTE10 + LPS improved the rats’ performance in TSNA% to 28.44 ± 0.5686%, counteracting the LPS-induced cognitive challenges. Finally, no significant alteration was noted with BHTE10 alone (26.56 ± 1.237%) compared to control rats.

### 3.4. BHTE Improved the Cholinergic Functions in LPS-Induced Rats

**Figure 5:**
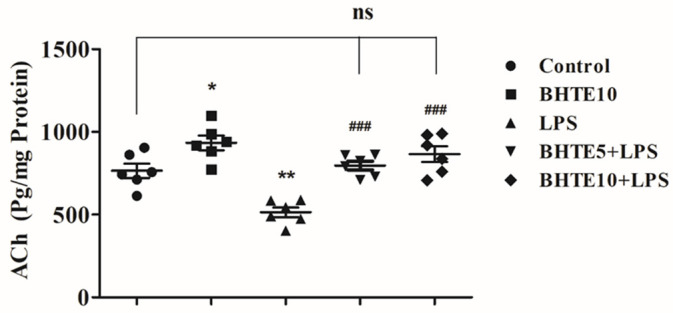
The influence of BHTE on ACh levels in LPS-induced rat brain. The results are reported as the mean ± SEM, with a sample size of n = 6. One-way ANOVA [F(4,25) = 16.77, *p* < 0.001], followed by Tukey–Kramer multiple comparisons test. * *p* < 0.05 and ** *p* < 0.01 vs. control; ns—not significant vs. control; ### *p* < 0.001 vs. LPS.

Among neurotransmitters, ACh plays a major role in various cognitive functions. In the present study, both BHTE and LPS treatments significantly altered brain ACh levels (pg/mg protein) in rats [F(4,25) = 16.77, *p* < 0.001] (Figure 5). The ACh level in the control animals was 765.1 ± 42.94. However, after LPS induction, ACh levels were significantly reduced by 32% (*p* < 0.01) to 514.2 ± 29.24. Fortunately, co-administration of BHTE with LPS showed a remarkable improvement in brain ACh levels. The BHTE5 + LPS treatment elevated ACh levels by 35% (*p* < 0.001) to 797.1 ± 26.60, and the BHTE10 + LPS treatment increased ACh levels by 40% (*p* < 0.001) to 865.7 ± 47.60. These results indicate an improvement in cholinergic function in LPS-induced brains. As expected, there was an elevation in ACh levels (933.9 ± 43.99) after consecutive thirty days of BHTE10 administrations, compared to control animals. This suggests that BHTE not only counteracts the negative effects of LPS on ACh levels but also enhances cholinergic function beyond baseline levels observed in control animals.

### 3.5. BHTE Protects LPS-Induced Neuroinflammation in Rats

**Figure 6:**
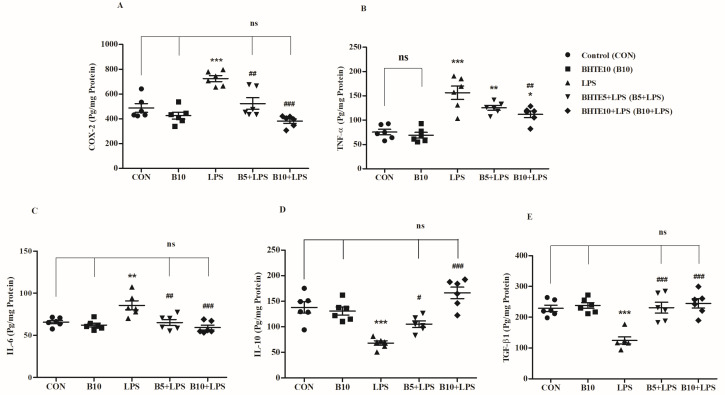
The impact of BHTE on inflammatory parameters such as (**A**) COX-2, (**B**) TNF-α, (**C**) IL-6, (**D**) IL-10, and (**E**) TGF-β1 in LPS-induced rats. The results are reported as the mean ± SEM, with a sample size of n = 6. One-way ANOVA [F(4,25) = 17.21, *p* < 0.001 for COX-2; F(4,25) = 20.26, *p* < 0.001 for TNF-α; F(4,25) = 9.131, *p* < 0.001 for IL-6; F(4,25) = 18.51, *p* < 0.001 for IL-10; and F(4,25) = 14.36, *p* < 0.001 for TGF-β1], followed by Tukey–Kramer multiple comparisons test. * *p* < 0.05, ** *p* < 0.01 and *** *p* < 0.001 vs. control; ns—not significant vs. control; # *p* < 0.05, ## *p* < 0.01 and ### *p* < 0.001 vs. LPS.

LPS is an endotoxin known as a potent pro-inflammatory agent and a major trigger for various inflammatory cascades. To evaluate the anti-neuroinflammatory capability of BHTE, the analysis targeted several pro-inflammatory markers, including COX-2, TNF-α, and IL-6, as well as anti-inflammatory markers such as IL-10 and TGF-β1 (Figure 6).

This study investigated the impact of BHTE and LPS treatments on COX-2 enzyme levels (pg/mg protein) in the brain tissues of rats. A significant alteration was observed among all the treatment groups [F(4,25) = 17.21, *p* < 0.001] (Figure 6A). LPS induction resulted in a 32% elevation (*p* < 0.001) of COX-2 levels, increasing to 724.7 ± 23.94 from the control level of 487.4 ± 35.08. This indicates a pronounced inflammatory response due to LPS. However, treatments with BHTE attenuated the LPS-induced elevation of COX-2 levels. The BHTE5 + LPS treatment reduced COX-2 levels by 27% (*p* < 0.01), bringing them down to 523.0 ± 47.11. Further, BHTE10 + LPS treatment showed an even more substantial reduction of 47% (*p* < 0.001), lowering COX-2 levels to 382.2 ± 18.42. Additionally, the administration of BHTE10 alone resulted in COX-2 levels of 425.4 ± 27.16, showing no significant changes compared to the control group.

The levels of the pro-inflammatory cytokine TNF-α (pg/mg protein) were significantly altered by BHTE and LPS treatments [F(4,25) = 20.26, *p* < 0.001] (Figure 6B). As expected, LPS stimulation resulted in a 51% increase (*p* < 0.001) in TNF-α levels, raising them to 156.3 ± 13.68 in the rats’ brains as compared to control level 75.42 ± 5.749. Among the treatments, only the higher dose, BHTE10 + LPS, successfully reduced TNF-α levels by 28% (*p* < 0.01) to 112.0 ± 6.691 compared to the LPS alone group. The TNF-α levels in the BHTE5 + LPS treatment were 125.2 ± 4.826. Additionally, BHTE10 alone resulted in TNF-α levels of 69.13 ± 5.813, similar to the control treatment.

The levels of the second targeted pro-inflammatory cytokine IL-6 (pg/mg protein) were also significantly affected by the treatments in this study [F(4,25) = 9.131, *p* < 0.001] (Figure 6C). Administration of LPS resulted in a 23% increase (*p* < 0.01) in brain IL-6 levels, raising them to 85.53 ± 5.382 from the control level of 65.75 ± 2.083. Both BHTE treatments successfully mitigated the LPS-induced elevation in IL-6 levels. The BHTE5 + LPS treatment reduced IL-6 levels by 23% (*p* < 0.01) to 65.12 ± 3.549, and the BHTE10 + LPS treatment further reduced IL-6 levels by 30% (*p* < 0.001) to 59.16 ± 2.848. Additionally, BHTE10 alone resulted in IL-6 levels of 62.09 ± 2.291.

On the other hand, the levels of the targeted anti-inflammatory cytokine IL-10 (pg/mg protein) were significantly modulated by all the treatments [F(4,25) = 18.51, *p* < 0.001] (Figure 6D). LPS administration significantly reduced IL-10 levels to 68.08 ± 4.294, a decrease of 51% compared to the control value of 138.0 ± 11.22 (*p* < 0.001). Notably, co-administration of BHTE5 + LPS counteracted this reduction, elevating IL-10 levels to 105.1 ± 6.255, an increase of 35% compared to LPS treatment alone (*p* < 0.01). Moreover, BHTE10 + LPS treatment significantly elevated IL-10 levels to 166.3 ± 11.27, a 59% increase compared to LPS induction. In the BHTE10 group, IL-10 levels were recorded at 130.8 ± 7.765.

As illustrated in Figure 6E, the levels of the second targeted anti-inflammatory marker, TGF-β1 (pg/mg protein), were also significantly influenced by both treatments [F(4,25) = 14.36, *p* < 0.001]. LPS administration reduced TGF-β1 levels by 45% to 124.5 ± 11.36, compared to the control level of 228.8 ± 10.66 (*p* < 0.001), indicating apparent inflammation in the brain. In contrast, co-administration of BHTE5 + LPS (231.2 ± 17.67; 46%; *p* < 0.001) and BHTE10 + LPS (245.1 ± 15.32; 49%; *p* < 0.001) significantly ameliorated the inflammatory deficits, as exhibited by restored TGF-β1 levels in the LPS-induced brain. Administration of BHTE10 alone resulted in TGF-β1 levels of 238.3 ± 9.379.

### 3.6. BHTE Protects LPS-Induced Neuronal Mitochondrial Dysfunction in Rats

**Figure 7:**
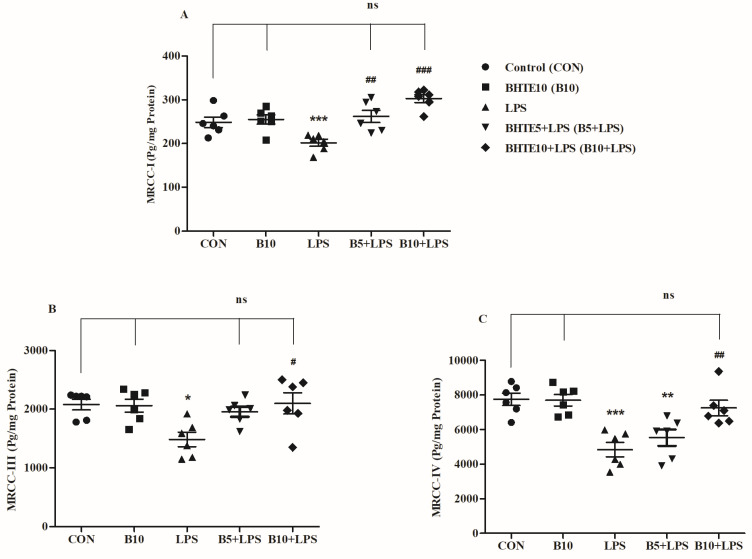
The effects of BHTE on mitochondrial parameters such as (**A**) MRCC-I, (**B**) MRCC-III, and (**C**) MRCC-IV in LPS-induced rats. The results are reported as the mean ± SEM, with a sample size of n = 6. One-way ANOVA [F(4,25) = 10.93, *p* < 0.001 for MRCC-I; F(4,25) = 4.400, *p* < 0.01 for MRCC-III; and F(4,25) = 11.12, *p* < 0.001 for MRCC-IV], followed by Tukey–Kramer multiple comparisons test. * *p* < 0.05, ** *p* < 0.01, and *** *p* < 0.001 vs. control; ns—not significant vs. control; # *p* < 0.05, ## *p* < 0.01, and ### *p* < 0.001 vs. LPS.

The enzyme complexes MRCC-I, MRCC-II, and MRCC-III, which are part of the series of inner mitochondrial membrane complexes and are considered to be involved in the process of neuronal degeneration, were analyzed in rat brain tissue following treatment with BHTE and LPS (Figure 7).

A significant statistical variation was observed in MRCC-I enzyme levels (pg/mg protein) in rat brains following BHTE and LPS treatment [F(4, 25) = 10.93, *p* < 0.001] (Figure 7A). Compared to the control group (248.5 ± 11.98), only the LPS-treated rats showed a significant decline in MRCC-I levels (201.8 ± 8.055; 19%; *p* < 0.001), while no considerable changes were noted in other treatment groups. However, concurrent administration of BHTE5 + LPS (262.1 ± 13.82; 23%; *p* < 0.01) and BHTE10 + LPS (302.7 ± 9.032; 33%; *p* < 0.001) successfully protected against the LPS-induced reduction in MRCC-I enzyme levels in the brain. The MRCC-I levels in the BHTE10 alone treatment group were 255.0 ± 10.63, showing no significant difference from the control.

When considering the effect of BHTE and LPS on MRCC-III levels (pg/mg protein), a significant alteration [F(4, 25) = 4.400, *p* < 0.01] was observed after treatment in rats (Figure 7B). Following LPS treatment alone, MRCC-III levels were significantly reduced (1485 ± 123.6; 28%; *p* < 0.05) compared to the control treatment (2080 ± 90.30). Only the higher dose of BHTE10 + LPS resulted in a considerable improvement in MRCC-III levels (2098 ± 179.5; 29%; *p* < 0.05) compared to the LPS-induced reduction. The MRCC-III levels for the BHTE10 alone and BHTE5 + LPS treatments were 2060 ± 112.0 and 1958 ± 86.46, respectively.

Similarly, the analysis of MRCC-IV enzyme levels (pg/mg protein) showed significant alterations among all the treatments [F(4, 25) = 11.12, *p* < 0.001] (Figure 7C). There was a significant reduction in MRCC-IV levels in the LPS-treated group (4837 ± 417.7; 37%; *p* < 0.001) compared to the control group (7747 ± 331.7). The BHTE5 + LPS treatment also showed a decline in MRCC-IV levels (5525 ± 47.30; 12%; *p* < 0.01) compared to the control group. However, the higher dose of BHTE10 + LPS elevated the enzyme levels to 7243 ± 448.6 (33%; *p* < 0.01) compared to the LPS-induced reduction. The enzyme levels in the BHTE10 alone group (7687 ± 331.7) were similar to those in the control group.

### 3.7. BHTE Preserves LPS-Induced Neuronal Apoptosis in Rats

**Figure 8:**
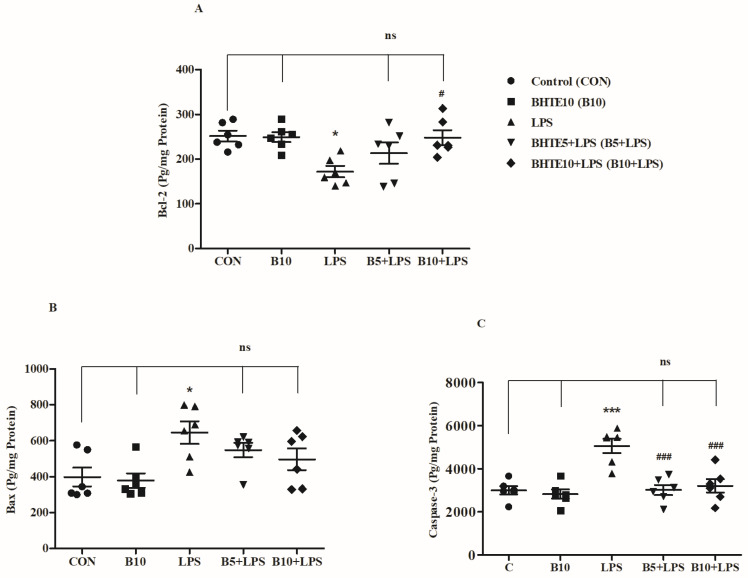
The impact of BHTE on apoptosis parameters including (**A**) Bcl-2, (**B**) Bax, and (**C**) caspase-3 in LPS-induced rats. The results are reported as the mean ± SEM, with a sample size of n = 6. One-way ANOVA [F(4,25) = 4.703, *p* < 0.01 for Bcl-2; F(4,25) = 4.512, *p* < 0.01 for Bax; and F(4,25) = 12.44, *p* < 0.001 for caspase-3], followed by Tukey–Kramer multiple comparisons test. * *p* < 0.05, and *** *p* < 0.001 vs. control; ns—not significant vs. control; # *p* < 0.05 and ### *p* < 0.001 vs. LPS.

To evidence the neuroprotective capability of BHTE against LPS-induced apoptotic insults in rat brain, analyses were conducted using the anti-apoptosis marker Bcl-2 and two pro-apoptosis markers, Bax and caspase-3 (Figure 8).

The levels of the anti-apoptotic protein Bcl-2 (pg/mg protein) significantly varied following treatment with LPS and BHTE [F(4,25) = 4.703, *p* < 0.01] (Figure 8A). In comparison to control animals (251.7 ± 11.74), LPS treatment reduced Bcl-2 levels to 172.0 ± 12.45, representing a 31% decrease (*p* < 0.05). Notably, treatment with BHTE10 + LPS (248.0 ± 16.80) significantly mitigated the LPS-induced reduction in Bcl-2 levels by 30% (*p* < 0.05). Bcl-2 levels for the BHTE5 + LPS and BHTE10 groups were 213.3 ± 23.81 and 249.3 ± 11.19, respectively.

Conversely, the levels of the pro-apoptotic protein Bax levels (pg/mg of protein) varied significantly after different treatments, as indicated by [F(4,25) = 4.512, *p* < 0.01] (Figure 8B). Specifically, a comparison between the groups revealed that only LPS administration significantly increased Bax levels (645.3 ± 61.58; 38%; *p* < 0.05) compared to the control group (397.9 ± 52.68). No significant changes in Bax levels were observed with other treatments when compared to either the control or LPS groups. The recorded Bax levels were 548.2 ± 39.69 for the BHTE5 + LPS group, 496.4 ± 60.59 for the BHTE10 + LPS group, and 378.3 ± 40.10 for the BHTE10 group.

Unlike Bax, another pro-apoptosis marker, caspase-3 levels (pg/mg of protein) were altered, when compared between two treatments. First, a comparison among all the groups revealed extensive variation [F(4,25) = 12.44, *p* < 0.001] in caspase-3 levels (Figure 8C). After LPS administration, it elevated the caspase-3 levels from 3000 ± 188.5 (control) until 5059 ± 332.1 (41%; *p* < 0.001). Also, the simultaneous treat of BHTE at two doses levels with LPS as BHTE5 + LPS (3017 ± 235.6; 40%; *p* < 0.001) and BHTE10 + LPS (3206 ± 311.0; 36%; *p* < 0.001) protected the effect of LPS on caspase-3 levels. With BHTE10, the caspase-3 level was 2830 ± 213.6.

### 3.8. BHTE Protects LPS-Induced Neuronal Oxidative Stress in Rats

**Figure 9:**
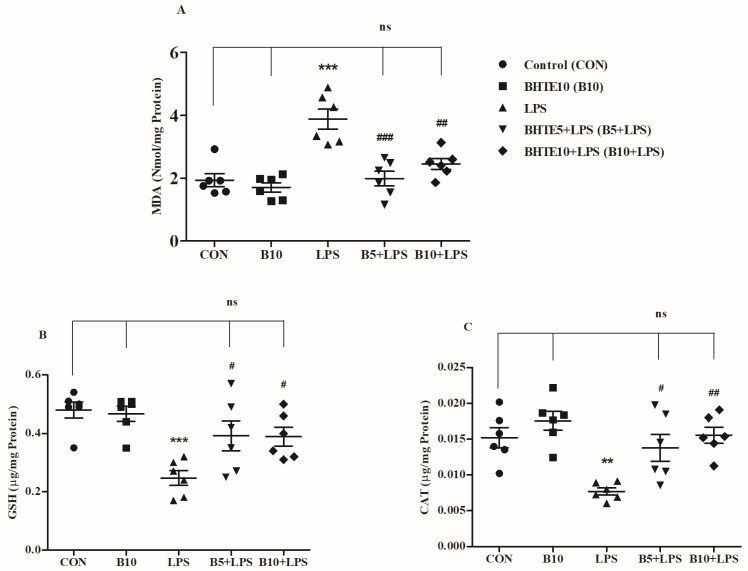
The effects of BHTE on oxidative parameters such as (**A**) MDA, (**B**) GSH, and (**C**) CAT in LPS-induced rats. The results are reported as the mean ± SEM, with a sample size of n = 6. One-way ANOVA [F(4,25) = 15.21, *p* < 0.001 for MDA; F(4,25) = 7.564, *p* < 0.001 for GSH; F(4,25) = 8.018, *p* < 0.001 for CAT], followed by Tukey–Kramer multiple comparisons test. ** *p* < 0.01 and *** *p* < 0.001 vs. control; ns—not significant vs. control; # *p* < 0.05, ## *p* < 0.01, and ### *p* < 0.001 vs. LPS.

The impact of BHTE and LPS treatments on oxidative-related markers was evaluated by measuring levels of an oxidative marker, MDA, and two antioxidant markers, GSH and catalase (Figure 9).

The brain MDA levels (nmol/mg protein) were significantly affected by BHTE and LPS treatments [F(4,25) = 15.21, *p* < 0.001] (Figure 9A). As anticipated, MDA levels increased markedly in LPS-treated rats, reaching 3.883 ± 0.3195 (50%; *p* < 0.001) compared to control rats (1.935 ± 0.2083). However, the lower dose treatment of BHTE5 + LPS significantly reduced MDA levels to 1.988 ± 0.2388 (48%; *p* < 0.001) relative to the LPS group. Similarly, BHTE10 + LPS treatment mitigated the LPS-induced rose in MDA levels, bringing them down to 2.455 ± 0.1725 (36%; *p* < 0.01). In rats treated with BHTE alone, MDA levels were 1.705 ± 0.1499.

The antioxidant marker GSH (µg/mg protein) levels were significantly altered by BHTE and LPS treatments [F(4,25) = 7.564, *p* < 0.001 for GSH] (Figure 9B). Compared to the control group (0.4800 ± 0.027), GSH levels were significantly reduced by 48% (0.2467 ± 0.0253; *p* < 0.001) following LPS administration. However, treatments with BHTE5 + LPS (0.3917 ± 0.0513; 37%; *p* < 0.05) and BHTE10 + LPS (0.3883 ± 0.0321; 36%; *p* < 0.05) effectively counteracted the LPS-induced reduction in GSH levels. In the group treated with BHTE10 alone, GSH levels were recorded at 0.4667 ± 0.0256.

The levels of another antioxidant, CAT (µg/mg protein), also showed significant changes in the brains of treated rats [F(4,25) = 8.018, *p* < 0.001] (Figure 9C). LPS treatment caused a significant decline in CAT levels to 0.0077 ± 0.0005 (49%; *p* < 0.01) compared to the control level of 0.0152 ± 0.0014. Interestingly, CAT levels increased significantly with BHTE treatments: BHTE5 + LPS elevated CAT levels to 0.0138 ± 0.0019 (44%; *p* < 0.05), and BHTE10 + LPS raised them to 0.0155 ± 0.0011 (50%; *p* < 0.01) compared to the LPS group. In the BHTE10 alone group, CAT levels were 0.0176 ± 0.0013.

### 3.9. Molecular Docking

**Figure 10:**
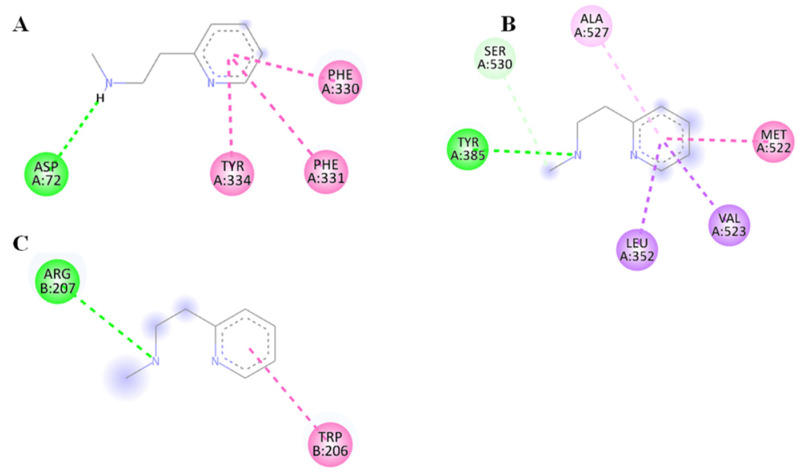
Lowest energy conformations of BHTE in the active site of AChE (**A**), COX-2 (**B**), and caspase-3 (**C**). The green and pink color dashed line represents hydrogen and hydrophobic interactions.

Our animal study results showed improvements in ACh levels and anti-inflammatory parameters, along with a decrease in pro-inflammatory parameters. The target fishing with the help of PharmMapper showed AChE, COX-2, and caspase-3 as the potential targets for the betahistine and hence were considered for molecular docking. The docking score of betahistine against AChE, COX-2, and caspase-3 was in the range of -4 to -6 kcal/mol. The binding mode analysis was performed to evaluate the molecular level interactions. It should be noted that in all three docked complexes first binding mode or the lowest energy conformation was considered for discussion. The binding mode analysis for BHTE-AChE complex showed hydrogen bond interaction with Asp72 and hydrophobic interactions with Phe330, Phe331, and Tyr334. The binding mode analysis of BHTE-COX-2 complex reveals hydrogen bond interaction with Tyr385 and hydrophobic interactions with Ser530, Leu352, Val523, Met522, and Ala527. Further the binding mode analysis of BHTE–caspase-3 complex shows hydrogen bond interaction with Arg207 and hydrophobic interactions with Trp206 (Figure 10).

## 4. Discussion

Our findings indicate that BHTE exerts neuroprotective effects in rats by mitigating cognitive impairments, cholinergic deficiencies, mitochondrial dysfunctions, neuronal apoptosis, and oxidative stress associated with LPS-induced neurotoxicity. Clinically, BHTE is primarily used to treat vertigo and Meniere’s disease by improving microcirculation in the inner ear [18]. Likewise, extensive evidence supports that targeting specific histamine receptor agonists, antagonists, and inverse agonists offers promising opportunities for harnessing the histaminergic system in the treatment of CNS disorders and the prevention of neurodegeneration [29,30,31]. Furthermore, abnormal glial activation and neuroinflammation are widely recognized as major contributors to the pathogenesis of neurodegenerative diseases, including AD and Parkinson’s disease (PD) [32]. Evidence suggests that glial activation and neuroinflammation can lead to oxidative stress, cholinergic deficiencies, mitochondrial dysfunctions, and neuronal apoptosis by producing excessive reactive oxygen species, impairing acetylcholine synthesis, disrupting mitochondrial energy production, and triggering cell death pathways, contributing to neurodegeneration [1,33]. In the present study, four doses of systemic LPS injection (1 mg/kg, i.p.) were used to construct a neuroinflammation-associated disease model, as several studies have demonstrated that LPS promotes neuroinflammation and neurodegeneration in animal studies [12,14,15,16,17,34]. According to the literature, evidence regarding BHTE’s neuroprotective effects, specifically in relation to neuroinflammation and other related mechanisms, is limited.

In this study, we confirmed LPS-induced cognitive impairments using three maze models (EPM, NOR, and Y-maze), each associated with different types of memory functions. Additionally, we evaluated the effects of BHTE on various memory parameters by administering two doses (5 and 10 mg/kg, p.o.) over 30 days. Our findings revealed that BHTE successfully reversed LPS-induced cognitive deficits across all maze models. Previously, few studies had assessed the reversal of cognitive deficits in various memory-related parameters. Among them, in a study with mice, BHTE enhanced the recall of forgotten memories in a NOR task after both one-week and one-month treatments by disinhibiting histamine release in the perirhinal cortex. Additionally, an extended human clinical trial demonstrated that BHTE was more effective in improving recall for difficult-to-remember items and in participants with initially lower performance [27]. Furthermore, the administration of BHTE reduced memory deficits caused by PTZ in the passive avoidance task and decreased depressive behavior in the forced swimming test [24]. The combined exposure to BHTE and lorcaserin (a 5HT_2C_ agonist) has been shown to enhance cognitive functions in multiple maze models, including NOR, Y-Maze, and object-in-place tasks, in rats with obesity-induced cognitive challenges [35]. Also, our preliminary experimental findings indicated that administering BHTE (5 and 10 mg/kg, p.o.) for 28 days mitigated the behavioral impairments caused by doxorubicin (DOX)-induced chemobrain. This was demonstrated by enhancements in EPM, NOR, and Y-Maze performance [28].

Initially, the EPM test was conducted to assess the effects of BHTE on the learning capability and retention capacity (memory) in rats, serving as an indicator of spatial memory. The TL was measured for each animal for two days, with higher TL values following LPS exposure indicating impaired learning capability and memory capacity [29]. Notably, administration of both doses of BHTE resulted in reduced TL values in a dose-dependent manner. Following the EPM test, the NOR tasks were performed, evaluating three parameters: ETFO, ETNO, and DI%. Collectively, these parameters are related to the recognition memory of rodents. Specifically, ETFO reflects the animal’s ability to recall previously encountered objects, ETNO indicates the animal’s ability to detect novelty and cognitive flexibility, and DI% reflects the animal’s ability to discriminate between familiar and novel objects [28,29,30]. Our results showed that LPS-induced deficits in recognition memory increased ETFO and ETNO while reducing DI%. However, BHTE treatments mitigated these impairments by reducing ETFO and ETNO and improving DI%. Finally, the Y-maze task was used to assess spatial memory and learning in rodents. Similar to the NOR test, three key parameters were evaluated: NEKA, NENA, and TSNA%. NEKA reflects the animal’s working memory and its ability to remember which arms have already been visited during the training session, NENA indicates spatial recognition memory and the animal’s ability to detect and explore new environments, and TSNA% reflects the animal’s exploratory behavior and preference for novelty, indicative of its recognition memory and cognitive flexibility [28,29,30]. Similar to previous tasks, BHTE administration protected against LPS-induced deficits in spatial memory and learning ability in animals.

Considering brain function, ACh is a vital neurotransmitter that plays a significant role in brain function, particularly in memory, behavior, and cognition. It is crucial for several stages of memory processing, such as encoding, attention, synaptic plasticity, consolidation, and retrieval. ACh supports both short-term, long-term memory, making it essential for cognitive functions related to memory [36,37]. Moreover, cholinergic functions supports recognition memory by enhancing encoding and retrieval, regulating neural activity in the hippocampus and prefrontal cortex, and improving synaptic plasticity, attention, and focus [38,39]. Additionally, the cholinergic network is also believed to enhance working memory by improving attention and focus, regulating neural activity in the prefrontal cortex, and fostering synaptic plasticity to facilitate flexible information updating and maintenance [38,40]. In the present study, BHTE administration elevated ACh levels in the brain tissues of LPS-challenged rats. This rise in ACh levels aligned with our maze test results, demonstrating that BHTE treatment successfully reversed various memory impairments and enhanced cognitive functions in the LPS-challenged model. It is well established that BHTE modulates neurotransmitters by blocking the H_3_R on presynaptic neurons, which regulates the release of histamine, dopamine, GABA, ACh, norepinephrine, and serotonin. This enhances neurotransmitter release, helping treat conditions like Ménière’s disease [41]. Additionally, BHTE has been shown to improve ACh levels in cases of DOX-induced decline in ACh levels in mouse brains [28].

Neuroinflammation in the CNS has dual roles, being both protective and harmful. Its mechanisms are crucial for creating effective neurological disorder treatments. Inflammation often precedes neurodegenerative diseases like AD and PD, playing a vital role in their development, supported by studies in cellular neuroscience and human genetics [42]. Considering pathological consequences, conditions such as chronic inflammation, excessive ROS production, the over release of glutamate by reactive glia, and inflammation-induced BBB weakening can lead to neuronal damage and death, ultimately contributing to cognitive decline and neurological deficits [43]. The current results indicated that LPS-induced neuroinflammation led to an increase in pro-inflammatory markers, such as COX-2, TNF-α, and IL-6, as well as decrease in anti-inflammatory markers, including IL-10 and TGF-β1. It is well established that COX-2 is crucial in neuroinflammation, facilitating the production of prostaglandins, which are central to the inflammatory process. In response to neuroinflammatory triggers, COX-2 levels increase in neurons and glial cells, leading to higher concentrations of pro-inflammatory prostaglandins. This contributes to neuronal damage, worsens inflammation, and may affect the progression of neurodegenerative diseases [44]. Targeting COX-2 can help manage inflammatory responses and reduce neuronal damage. Presently, treatment with BHTE at doses of 5 and 10 mg/kg (p.o.) effectively maintained COX-2 levels in brain tissue that were increased due to LPS administration. Further, prostaglandins generated by COX-2 can enhance the production of pro-inflammatory cytokines including TNF-α and IL-6 via multiple signaling pathways, which activate NF-κB and other transcription factors. These elevated cytokines can further increase COX-2 expression, forming a positive feedback loop that intensifies and sustains the inflammatory response [45]. In neuroinflammation, this mechanism can cause considerable neuronal damage and promote the progression of neurodegenerative diseases [45,46]. The neuroprotective effects of BHTE were presently evidenced by a significant reduction in TNF-α and IL-6 levels, as well as COX-2 inhibition, in the brains of LPS-treated rats. These findings suggest that BHTE may effectively protect against LPS-induced neuroinflammation.

In neuroinflammation, pro-inflammatory cytokines contribute to neuronal damage and disease progression, whereas anti-inflammatory cytokines are essential for mitigating these effects and supporting neuroprotection and tissue repair. IL-10 and TGF-β1 are prominent anti-inflammatory cytokines in this context [47]. IL-10, produced by microglia, astrocytes, and peripheral immune cells, inhibits the production of pro-inflammatory cytokines such as TNF-α, IL-1β, and IL-6 by interfering with transcription factors like NF-κB. This action helps reduce inflammation and prevent excessive neuronal damage [47,48]. Similarly, TGF-β1 suppresses the production of pro-inflammatory cytokines, chemokines, and other inflammatory molecules, limiting the recruitment and activation of immune cells at the inflammation site [47,49]. Our current results showed that BHTE has anti-inflammatory effects in rat brains by elevating the levels of IL-10 and TGF-β1. This action helps mitigate LPS-induced inflammation, leading to a reduction in the levels of these cytokines.

MRCCs are essential enzyme complexes in the inner mitochondrial membrane that drive cellular respiration and energy production. They enable oxidative phosphorylation, where electrons are transferred through carriers, ultimately generating ATP, the primary energy sources of cells [50]. In neurons, these functions are particularly critical due to their high energy demands, necessitating ATP for maintaining membrane potential, action potential propagation, synaptic transmission, calcium homeostasis, neuroplasticity, and cellular repair [51]. Complexes I, III, and IV have especially more attention in the context of neurodegeneration, as they are vital for maintaining cellular energy balance and reducing oxidative stress. Dysfunction in these complexes can lead to harmful effects that contribute to neurodegenerative diseases [52,53]. In detail, complex I transfers electrons from NADH to ubiquinone and pumps protons across the inner mitochondrial membrane; impaired complex I reduces ATP production and increases ROS, causing oxidative damage to neuronal proteins, lipids, and DNA. Following, complex III transfers electrons from ubiquinol to cytochrome c and pumps protons to sustain the electrochemical gradient; dysfunction here impairs ATP synthesis, increases superoxide production, and causes oxidative stress and mitochondrial damage, with mutations linked to AD and Leigh syndrome [53]. In extension, complex IV transfers electrons from cytochrome c to oxygen, reducing it to water and pumping protons to generate the electrochemical gradient; impaired complex IV reduces ATP production, disrupts energy metabolism, and increases ROS production, leading to oxidative damage. Reduced complex IV activity is observed in AD patients’ brains, contributing to neuronal dysfunction and cell death [52,53,54]. Additionally, mitochondria are involved in inflammatory responses; blocking complex I in pro-inflammatory microglia protects the CNS against neurotoxic damage and improves outcomes in animal models [55]. Recent studies show that inhibiting mitochondrial complexes, particularly complex IV, amplifies LPS-induced IL-6 levels and alters the IL-6/TNF-α ratio in human blood leukocytes [56]. Other study indicated that LPS-induced neuroinflammation leads to mitochondrial functional deficits by affecting the mitochondrial outer membrane protein Mitofusin2 [57]. Our study demonstrated LPS-induced neurotoxicity in rat brains by showing decreased levels of MRCC-I, II, and III after four systemic injections. Notably, thirty days of BHTE administration improved LPS-induced mitochondrial dysfunction by increasing the levels of MRCC-I, II, and III at a high dose (10 mg/kg, p.o) in rat brains.

Apoptosis, a programmed cell death, is crucial for tissue development and maintenance. In neurons, it removes excess cells for proper neural circuit formation. However, improper activation of apoptotic pathways can lead to neurodegeneration. The intrinsic (mitochondrial) pathway is triggered by internal stress signals like DNA damage and oxidative stress, while the extrinsic (death receptor) pathway is initiated by external ligands binding to death receptors like Fas (CD95) and tumor necrosis factor receptor (TNFR) [58]. Further, apoptosis in neurons can be activated by several factors, including inadequate neurotrophic support, over activation of glutamate receptors leading to calcium influx, oxidative stress, and metabolic disturbances. Key mitochondrial changes during this process include increased production of reactive oxygen species, membrane pore opening, and cytochrome c release [59]. In the present study, the focus was on targeting an anti-apoptotic protein (Bcl-2) and two pro-apoptotic proteins (Bax and caspase-3) to elucidate the effects of BHTE on LPS-induced apoptosis. Among them, BAX is a pro-apoptotic protein in the BCL-2 family that promotes apoptosis by relocating to the mitochondria in response to apoptotic signals. This relocation causes the mitochondrial outer membrane to become permeable, allowing for the release of cytochrome c, which leads to activating the caspase cascade [60]. Further, caspase-3 is a key executioner caspase involved in the final phase of apoptosis. It cleaves several cellular substrates, causing typical apoptotic changes like DNA fragmentation and cell shrinkage. Functioning downstream of the BAX-induced cytochrome c release and apoptosome formation, caspase-3 breaks down cellular structures, leading to cell death [61]. On the other hand, BCL-2 is vital for preventing apoptosis in neurons by preserving mitochondrial integrity. It prevents the permeabilization of the outer mitochondrial membrane and inhibits the release of cytochrome c, thereby blocking the activation of downstream caspases needed for cell death. By counteracting pro-apoptotic proteins like BAX, BCL-2 promotes cell survival and maintains neuronal health [62]. The present results indicated that following LPS induction, Bax and caspase-3 levels were elevated in the brain. However, treatment with BHTE effectively reduced only the impact of caspase-3. In contrast, Bcl-2 levels decreased due to LPS induction, but administering a higher dose of BHTE (10 mg/kg, p.o.) significantly counteracted these effects.

Together, oxidative stress contributes to various neurodegenerative mechanisms, including neuroinflammation, mitochondrial dysfunction, and neuronal apoptosis. It induces neuroinflammation by activating glial cells and releasing inflammatory mediators [63]. It also leads to mitochondrial dysfunction by damaging mitochondrial components, resulting in reduced ATP production and increased ROS levels. Furthermore, oxidative stress initiates apoptosis by activating pro-apoptotic proteins and the caspase cascade, culminating in the death of neurons [58,63]. In the present study, LPS induction led to a rise in MDA levels and a reduction in catalase and GSH levels, indicating oxidative stress in brain tissues. MDA serves as a marker of oxidative stress, being a final product of lipid peroxidation that occurs when ROS target polyunsaturated fatty acids in cell membranes. Additionally, MDA can form adducts with DNA and proteins, impairing their function and contributing to various pathological conditions, including inflammation, aging, and neurodegenerative diseases [63,64]. In neurons, antioxidant systems serve as critical defense mechanisms that counteract oxidative insults. These systems, including enzymes like catalase and molecules like GSH, neutralize ROS and repair oxidative damage. Regarding enzyme catalase decomposes hydrogen peroxide into water and oxygen. This process prevents the formation of harmful hydroxyl radicals and protects cellular components like DNA, proteins, and lipids from oxidative damage, thus preserving cellular integrity and function [63]. Further, GSH, a cellular antioxidant consisting of glutamine, cysteine, and glycine, plays a key role in managing oxidative stress. It neutralizes ROS such as hydroxyl and superoxide radicals and supports glutathione peroxidase in reducing hydrogen peroxide and organic peroxides [65]. When antioxidant defenses are compromised, oxidative damage can build up in neurons, causing cellular dysfunction and contributing to neurodegenerative diseases. Notably, BHTE mitigated LPS-induced oxidative stress by lowering MDA levels and enhancing antioxidant defenses through increased GSH activity and catalase levels in brain tissues.

In order to find the molecular level mechanism, BHTE was subjected to the target fishing resulting into the identification of AChE, COX-2, and caspase-3 as the potential targets. Further the molecular level mechanism of BHTE against the potential targets was demonstrated using molecular docking. The results from the molecular docking showed good binding affinity of BHTE against AChE, COX-2, and caspase-3. Further, the top three low energy conformations were considered for binding mode analysis, and it was observed that the lowest energy conformation showed interactions with key residues. In the case of the AChE-BHTE docked complex, hydrogen bond interaction was observed with Asp72, and the hydrophobic interaction was noticed with Phe330, Phe331, and Tyr334. It should be noted that Asp72 is an important residue in the catalysis of ACh, and Tyr334 plays important role in substrate binding. Further, Phe330 and Phe331 are involved in the formation of the anionic site of AChE enzyme [66]. For the COX-2-BHTE docked complex, the hydrogen bond interaction was observed with Tyr385 located at the bent of the hydrophobic tunnel. Further, the hydrophobic interaction was observed with Leu352, Met522, Val523, and Ala527. It should be noted that the residues involved in hydrophobic interactions are important component of hydrophobic tunnel through which substrate arachidonic acid accesses the oxygenation site. For the case of the caspase-3–BHTE docked complex, the hydrogen and hydrophobic interactions were as observed for the co-crystallized ligand. Therefore, considering the results from animal studies and molecular docking, it can be safely stated that the observed efficacy of the BHTE is also because of a potential reduction in the activity of AChE, COX-2, and caspase-3 in addition to their expression levels.

## 5. Conclusions

In summary, this study demonstrates the notable neuroprotective effects of BHTE in a rat model of LPS-induced neurotoxicity. A 30-day oral pre-treatment with BHTE at doses of 5 or 10 mg/kg effectively countered cognitive impairments and restored acetylcholine (ACh) levels diminished by LPS exposure. BHTE’s beneficial impact included lowering neuroinflammatory markers (COX-2, TNF-α, IL-6) and oxidative stress, while boosting anti-inflammatory markers (IL-10, TGF-β1), mitochondrial enzyme activities (MRCC-I, II, and IV), and antioxidant defenses (GSH and catalase). Furthermore, BHTE increased the anti-apoptotic protein Bcl-2 and reduced pro-apoptotic proteins (Bax, caspase-3) and oxidative stress markers (MDA). Molecular modeling reinforced these findings by showing BHTE’s effective binding with key residues of AChE, COX-2, and caspase-3. Overall, these findings underscore BHTE’s potential as a treatment for CNS-related disorders by enhancing cholinergic and mitochondrial functions and protecting against neuroinflammation, cellular apoptosis, and oxidative stress.

## Data Availability

The data presented in this study are available from the corresponding author upon reasonable request. The data are not publicly available due to privacy issues.
